# Dendritic and Spine Heterogeneity of von Economo Neurons in the Human Cingulate Cortex

**DOI:** 10.3389/fnsyn.2020.00025

**Published:** 2020-07-08

**Authors:** Nivaldo D. Correa-Júnior, Josué Renner, Francisco Fuentealba-Villarroel, Arlete Hilbig, Alberto A. Rasia-Filho

**Affiliations:** ^1^Graduate Program in Biosciences, Universidade Federal de Ciências da Saúde de Porto Alegre (UFCSPA), Porto Alegre, Brazil; ^2^Laboratory of Morphology and Physiology, Department of Basic Sciences/Physiology, Universidade Federal de Ciências da Saúde de Porto Alegre, Porto Alegre, Brazil; ^3^Graduate Program in Neuroscience, Universidade Federal do Rio Grande do Sul, Porto Alegre, Brazil; ^4^Department of Medical Clinics/Neurology, Universidade Federal de Ciências da Saúde de Porto Alegre, Porto Alegre, Brazil

**Keywords:** dendritic spines, Golgi method, human brain/cytology, neocortical layer V, modified pyramidal neurons, paralimbic cortex, 3D reconstruction

## Abstract

The human cingulate cortex (CC), included in the paralimbic cortex, participates in emotion, visceral responses, attention, cognition, and social behaviors. The CC has spindle-shaped/fusiform cell body neurons in its layer V, the von Economo neurons (VENs). VENs have further developed in primates, and the characterization of human VENs can benefit from the detailed descriptions of the shape of dendrites and spines. Here, we advance this issue and studied VENs in the anterior and midcingulate cortex from four neurologically normal adult subjects. We used the thionin technique and the adapted “single-section” Golgi method for light microscopy. Three-dimensional (3D) reconstructions were carried out for the visualization of Golgi-impregnated VENs’ cell body, ascending and descending dendrites, and collateral branches. We also looked for the presence, density, and shape of spines from proximal to distal dendrites. These neurons have a similar aspect for the soma, but features of spiny dendrites evidenced a morphological heterogeneity of CC VENs. Only for the description of this *continuum* of shapes, we labeled the most common feature as VEN 1, which has main dendritic shafts but few branches and sparse spines. VEN 2 shows an intermediate aspect, whereas VEN 3 displays the most profuse dendritic ramification and more spines with varied shapes from proximal to distal branches. Morphometric data exemplify the dendritic features of these cells. The heterogeneity of the dendritic architecture and spines suggests additional functional implications for the synaptic and information processing in VENs in integrated networks of normal and, possibly, neurological/psychiatric conditions involving the human CC.

## Introduction

The cingulate cortex (CC) is part of the “proisocortex” in the paralimbic cortex, which is phylogenetically older than the neocortex along with the evolution of the mammalian brain (Braak, 1979; Kolb and Whishaw, 2015; Pandya et al., 2015). The human CC begins adjacent to the genu of the corpus callosum corresponding to the cortical area 24 of Brodmann and the agranular anterior limbic area with the subdivision area 33. The CC extends from the precingulate to the area *limitans*, numbered areas 36–38 by von Economo and Koskinas (Triarhou, 2009; see further subdivisions and classification in Vogt, 2015; functional and connectivity-based organization in Cauda et al., 2013; Glasser et al., 2016). The anterior cingulate cortex (ACC), initially considered a component of the superior limbic lobe (Talairach and Tournoux, 1993; Triarhou, 2009), is adjacent to the functionally distinct midcingulate cortex (MCC) (Vogt, 2015). Anterograde and retrograde tracing data showed CC connections with the prefrontal, premotor and motor, orbitofrontal, insular, and anterior temporal cortex as well as with some amygdaloid, hypothalamic, and thalamic nuclei and the periaqueductal gray matter in primates (Nieuwenhuys et al., 1988; Craig, 2002; Watson, 2006; Pandya et al., 2015 and references therein). In our species, the parietal cortex and the ventral striatum are also included (Vogt, 2015). The CC integrates specialized networks for attentional processes/executive functions with sensory, high-order associative and limbic brain areas. In this regard, the human CC elaborates focused attention for problem-solving, goal-directed, and exploratory behaviors; long-term memory and cognitive processing; premotor planning with motivational features; social awareness and emotions as love, trust, empathy, deception, guilt, and fear; feeding and aggression; and sympathetic and parasympathetic responses to modulate heart rate and arterial pressure, respiratory, and gastrointestinal responses (Allman et al., 2001, 2010, 2011a; Watson, 2006; Watson et al., 2006; Xuan et al., 2016; Rangwala et al., 2017 and references therein).

The human CC has a specialized neuronal type with an elongated “spindle-shaped” or rod-shaped cell body in its layer V (Nimchinsky et al., 1995). These cells, named von Economo neurons (VENs; Triarhou, 2009; Seeley et al., 2012), differ morphologically from the adjacent layer V pyramidal neurons (Feldman, 1984; Braak and Braak, 1985) and are larger than small-layer VI fusiform neurons (Nimchinsky et al., 1999). Congruent results with Nissl/thionin staining, Neu-N neuronal nuclear antigen, and immunoreactivity for functional biomarkers (Nimchinsky et al., 1995; Fajardo et al., 2008; Stimpson et al., 2011) identified VENs with vertically oriented fusiform soma and two main perpendicularly oriented primary dendrites emerging from opposite extremes, one directed to the superficial cortical layers and another directed to the white matter (Nimchinsky et al., 1995, 1999; Fajardo et al., 2008; Raghanti et al., 2015; González-Acosta et al., 2018), with few and usually short side braches (Watson, 2006). VENs do not express immunoreactivity for interneuron markers (i.e., parvalbumin, calbindin, or calretinin) but project axons to the subcortical white matter, some entering into the cingulum bundle (Nimchinsky et al., 1995), toward the brainstem or spinal cord regions (Cobos and Seeley, 2015).

The CC VENs have a characteristic phylogenetic and ontogenetic development (Allman et al., 2011a, b; Cauda et al., 2014). The ACC VENs evolved with a clustering pattern in our species and our closest relative living primates, the great apes (Nimchinsky et al., 1999; Allman et al., 2011b; Raghanti et al., 2015). VENs correspond to only 5.6% compared to the number of layer V pyramidal neurons (Nimchinsky et al., 1999) and approximately 3% of all neurons in layer V in the human ACC (Fajardo et al., 2008). Most human VENs mature along with the postnatal brain development (Allman et al., 2010, 2011a; Butti et al., 2013; Raghanti et al., 2015). Indeed, VENs are rare during gestation, and numbers increase during the first 8 months after birth, decrease and reach the adult number at 4–8 years (Allman et al., 2010, 2011a), and remain constant throughout aging in individuals with average cognition, but are comparatively more numerous in individuals (≥ age 80) who show outstanding memory abilities (Gefen et al., 2018).

The pattern of dendritic branching and the presence of pleomorphic spines provide relevant morphological criteria for the classification of neurons (Ramón y Cajal, 1909-1911; Ramón-Moliner, 1962; Braak, 1980; Gabbott et al., 1997; Vásquez et al., 2018). This is an important issue because the dendritic architecture relates to the biophysical properties of the neuron, the membrane available for contacts and integration of excitatory and inhibitory inputs, and the establishment of spatiotemporal domains for the synaptic computations (Spruston, 2008; Spruston et al., 2013; Rollenhagen and Lübke, 2016). Furthermore, dendritic spines are specialized postsynaptic units for most excitatory inputs, increasing the density of synapses in each cell as well as the possibilities for modulation and plasticity of information transmission (Bourne and Harris, 2008; Rochefort and Konnerth, 2012; Spruston et al., 2013; Brusco et al., 2014; Stewart et al., 2014; Woolfrey and Srivastava, 2016). Spines show varied shapes and sizes whose complexity is more evident in the human brain (Ramón y Cajal, 1909-1911; Yuste, 2013; Dall’Oglio et al., 2015). Dendritic spines can have various region-specific and neuron-specific functional implications (Fiala et al., 2002; Hayashi-Takagi et al., 2015; Nakahata and Yasuda, 2018) and show structural changes in neurological and psychiatric disorders (Penzes et al., 2011; Herms and Dorostkar, 2016).

There are few studies describing the dendritic architecture and spine diversity of layer V VENs in the human CC. For example, VENs were reported as fusiform cells with sparse dendritic trees and symmetric apical and basal branches with fewer spines than pyramidal neurons (Watson et al., 2006). Two types of spiny VENs in the human ACC with different dendritic lengths were defined, the small VENs with a total dendritic length of 1,500–2,500 μm and the large ones with 5,000–6,000 μm (Banovac et al., 2019). We obtained further morphological data to depict the heterogeneity of the VENs in the human CC. The VENs in layer V were identified by the thionin technique and further visualized by three-dimensional (3D) reconstruction of Golgi-impregnated neurons. Although having a spindle-shaped cell body with similar longitudinal length, CC VENs show heterogeneity in their dendritic branching pattern, ranging in a morphological *continuum* from sparsely branched to more extensively ramified cells. The 3D images evidenced additional differences in the distribution, density, and shapes of dendritic spines in these VENs. The morphological and likely functional implications are provided below.

## Materials and Methods

### Subjects

The subjects were two men and two women. Age, *postmortem* interval, cause of death, and type of tissue fixation are shown in Table 1. All ethical and legal procedures were carried out in accordance with the international regulatory standards based on the Helsinki Declaration of 1964. Written informed consent for brain donation was obtained with a next of kin during an autopsy at the morgue. The privacy rights of subjects were always observed. The Brazilian Ethics Committee from the Federal University of Health Sciences of Porto Alegre (UFCSPA; #62336116.6.0000.5345 and 18718719.7.0000.5345) approved this study.

**TABLE 1:** Characteristics of the human cases.

Cases	Age (years)	Sex	PMI (hours)	IQCODE	Cause of death	Fixation	Technique
1	91	F	≥ 6:00	1.32	Pneumonia	Immersion	Thionin/Golgi
2	62	F	≥ 6:00	3.00	Undetermined	Immersion	Thionin/Golgi
3	79	M	≥ 6:00	3.15	Cardiac Arrest	Immersion	Thionin/Golgi
4	49	M	≥ 6:00	3.00	Undetermined	Immersion	Thionin/Golgi

*PMI, post mortem interval; F, female; M, male.*

Donors’ clinical and comorbidity information was also obtained by interviewing a next of kin at the morgue. Subjects were reportedly healthy neurologically and psychiatrically, had no previous neurosurgical interventions, and were rated screened for cognitive decline using the “Informant Questionnaire on Cognitive Decline in the Elderly” (IQCODE; Neto et al., 2017). This is a validated interview procedure for which cutoff point scores of ≥ 3.27 or 3.48 are considered indicative of dementia in the Brazilian population (Sanchez and Lourenço, 2009; Carrabba et al., 2015). Only cases below these edge values were studied (Table 1). Besides, brain tissue from each subject was analyzed histologically and immunohistochemically by a neurologist/neuropathologist (AH) to confirm the absence of common vascular lesions or neurodegenerative disorders (data not shown).

### Tissue Processing for the Thionin Staining

All brains were kept immersed in 10% laboratory-grade, unbuffered formaldehyde solution at room temperature (RT) for approximately 5 years before the present procedure. The medial border of the cerebral hemisphere, the corpus callosum, and the characteristic aspect of the CC served as anatomical references (Talairach and Tournoux, 1993). The left hemisphere CC was studied from −36.0 mm to 5.4 mm from anterior to posterior position related to the midpoint of the anterior commissure (plates 5–26 according to Mai et al., 2008).

From each brain, tissue blocks containing the CC were sectioned and postfixed at RT for 30 days using a phosphate buffer solution (PBS, 0.1 M, pH = 7.4) with 4% formaldehyde and 1.5% picric acid. Then, the samples were sectioned in the coronal plane with a vibrating microtome (1000S; Leica, Germany) in an alternating fashion. One series was sectioned at 50 μm for the thionin technique. The other series was sectioned at 200 μm for the Golgi method.

**FIGURE 1:**
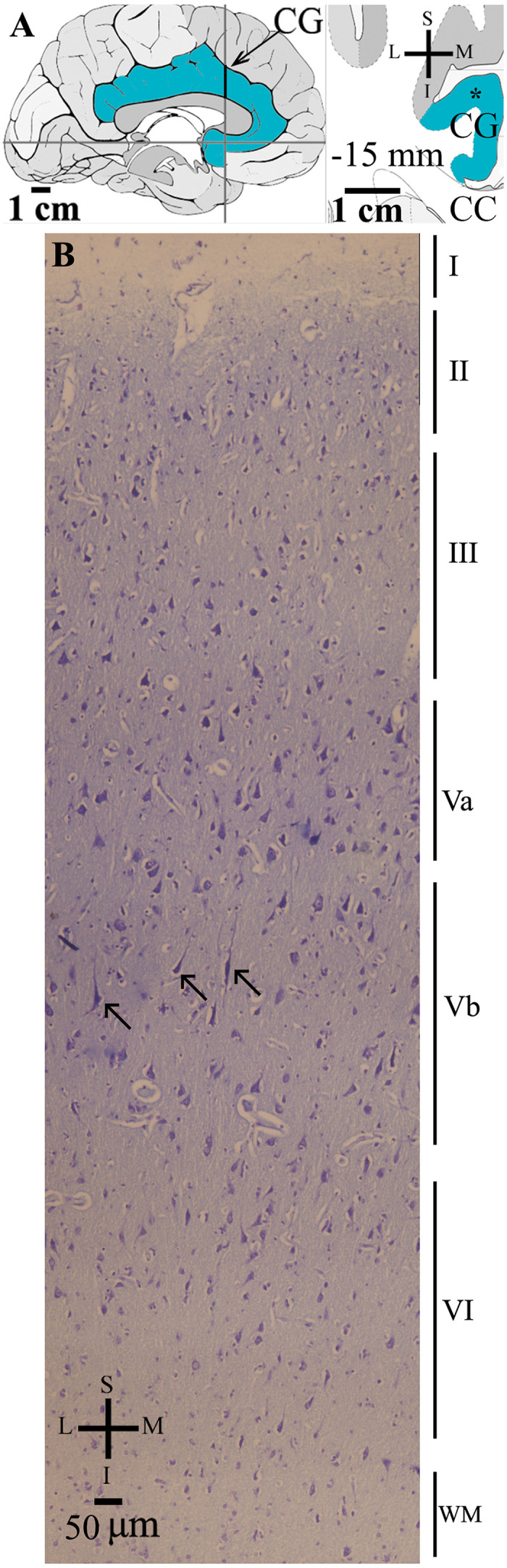
**(A)** Left: Schematic drawing of the medial view of the human brain showing the location of the cingulate gyrus (CG, highlighted in blue), in this case 15 mm anterior to the midpoint of the anterior commissure. Right: Higher-magnification drawing of the CG. The asterisk represents the approximate location of the brain section used for the study of the cortical cytoarchitecture stained in **(B)**. CC, corpus callosum. Adapted from Mai et al. (2008). **(B)** Photomicrograph of thionin-stained cells in layers I–VI of the human cingulate cortex. Note the characteristic absence of layer IV and a von Economo neuron (indicated by an arrow) with an elongated spindle-shaped cell body and two primary dendrites in layer Vb. WM, white matter. Coordinates: I, inferior; L, lateral; M, medial; S, superior.

**FIGURE 2:**
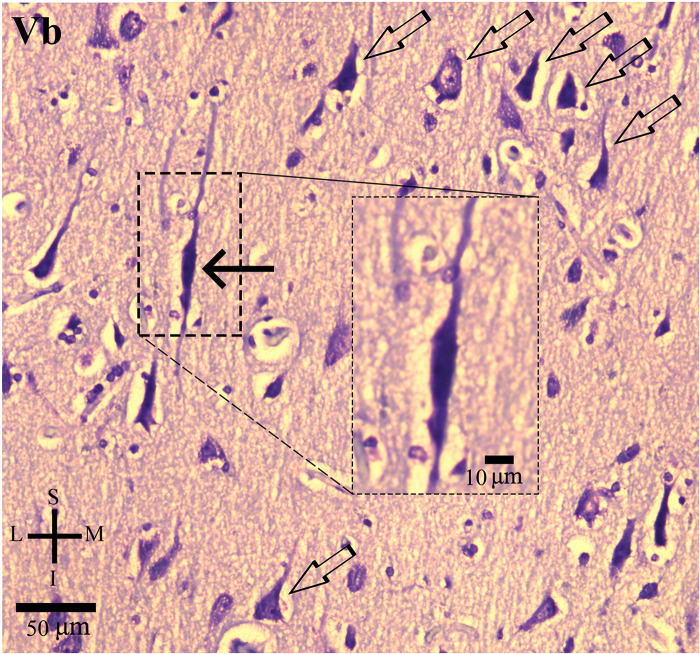
Photomicrograph of thionin-stained cells in the layer Vb of the human cingulate cortex as presented in Figure 1. The von Economo neurons (VEN, black solid arrow) are intermingled with pyramidal neurons (open arrows). Higher magnification of VEN (box). Note the characteristic elongated spindle-like cell body and two primary dendrites, one with an ascending direction and another with a descending aspect. Coordinates: I, inferior; L, lateral; M, medial; S, superior.

The thionin staining was used to identify the different cells and layers in the CC (Figures 1, 2). Staining began by placing serial sections from each tissue block on gelatin-coated slides and left to dry at RT for 1 day. Afterward, the slides were (1) immersed in a 4% formaldehyde in PBS for 1 week at 4°C protected from light; (2) dried for 1 day at RT and placed in a 70% ethanol solution for another day; (3) immersed in solutions of increasing concentrations of ethanol; (4) cleared in absolute xylene; (5) immersed in decreasing solutions of ethanol and washed in distilled water; (6) immersed in a solution of 0.25% thionin (Merck, Germany) for 3 min; (7) immersed again in solutions of increasing ethanol concentration; (8) dipped in a solution of 95% ethanol with 1% acetic acid and absolute xylene; and (9) mounted with synthetic balsam (Soldan, Brazil) and coverslipped (Dall’Oglio et al., 2013).

### The Golgi Method and the 3D Reconstruction Procedure

The “single-section” Golgi method was adapted to provide reliable results for the neuronal cell body and the dendritic and spines features in long-term fixed human brains (Dall’Oglio et al., 2010). The consistency of the present procedure is the same that served for previous characterization of neurons and dendritic spines in subcortical and cortical human brain areas (e.g., the medial and cortical amygdaloid nuclei and the CA3 hippocampal area; Dall’Oglio et al., 2013, 2015; Reberger et al., 2018; Vásquez et al., 2018).

The CC sections were kept for 3 days immersed in the post-fixation solution at RT. Afterward, sections were (1) rinsed in PBS and transferred to a solution of 0.1% osmium tetroxide (Sigma Chemicals Co., United States) in PBS for 20 min; (2) rinsed in PBS and immersed in 3% potassium dichromate (Merck) at 4°C in the dark for 2 days; (3) rinsed again in distilled water, “sandwiched” between coverslips, and placed in a solution of 1.5% silver nitrate (Merck) at RT in the dark for 1 day; (4) washed in distilled water; (5) placed on gelatin-coated histological slides, dried at RT, and dehydrated in an ascending series of ethanol (from 70 to 100% for 3 min each); (6) cleared in ethanol and absolute xylene; and (7) covered with non-acidic synthetic balsam (refractive index = 1.518–1.521, Permount Mounting Medium, EMS, USA or similar product, Soldan, Brazil) and coverslips.

We used the following including criteria to select neurons for analysis: (1) have cell bodies located within the boundaries of the ACC and MCC and in the cortical layer V; (2) have the cell body shape and primary dendrites characteristic of VENs; (3) be isolated from neighboring cells to avoid “tangled” dendrites; (4) have dendrites with defined borders and, as much as possible, tapering after branching or at distal locations; and (5) have dendritic spines distinguishable from the background.

The general morphology of selected neurons was studied at ×260 (using an objective plan apochromatic lens UPlanSApo 0.6 NA, Olympus, Japan) in a light microscope (Olympus BX-61, Japan) equipped with a z-stepping motor and coupled to a CCDDP72 high-performance camera (Olympus, Japan). Each image was acquired after advancing 0.5 μm for each z stack, under high resolution (1360 × 1024 pixels), and submitted to dynamic deconvolution using the Image-Pro Plus 7.0 software (Media Cybernetics, United States) during the acquisition process (Dall’Oglio et al., 2013; Reberger et al., 2018). Files were recorded as.TIFF files. The selected images were converted to 8-bit monochromatic pictures before processing.

**FIGURE 3:**
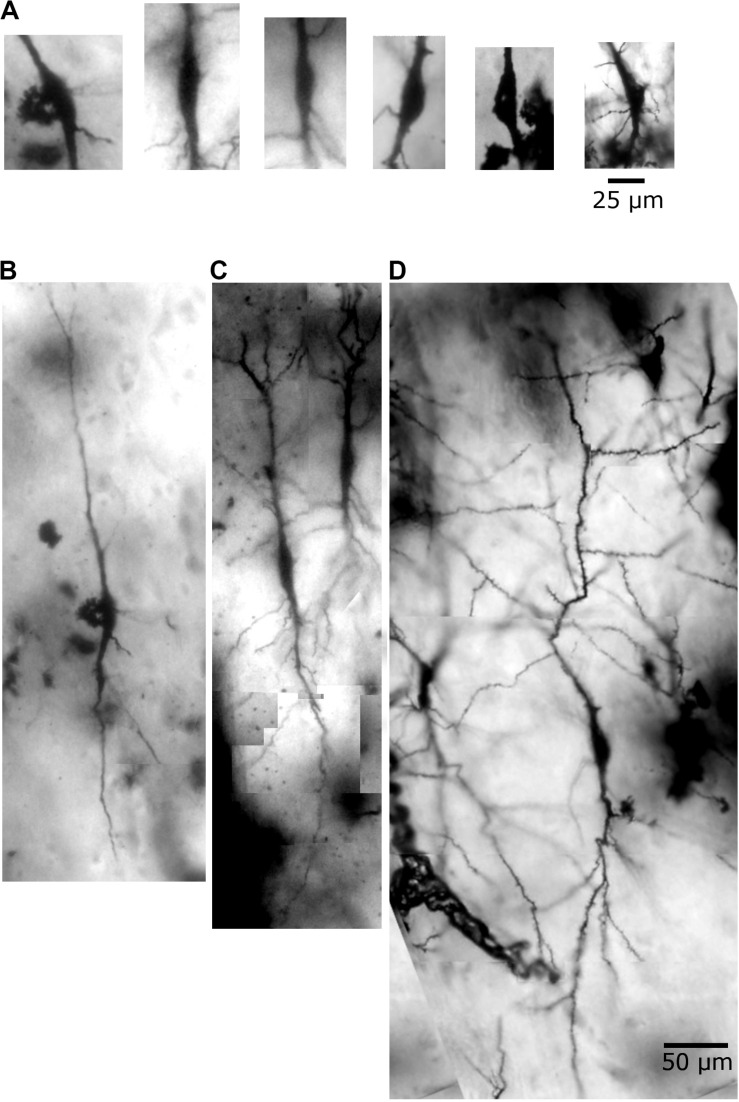
Human *postmortem* Golgi-impregnated von Economo neurons (VENs) in the cingulate cortex as observed in bright-field microscopy. **(A)** Cell body aspect of VENs evidencing a spindle-shaped soma with vertically oriented main primary dendritic shafts. **(B–D)** Golgi-impregnated VENs partially showing the dendritic ramification and spatial orientation. These VENs are shown reconstructed in Figures 4–8 and Supplementary Videos 1–3. Dendritic spines are not quite visible at this magnification. Image adjustment of contrast and brightness made with Photoshop CS3 (Adobe Systems, Inc., United States).

We first performed a two-dimensional (2D) reconstruction of Golgi-impregnated neurons by summing microscopic images at sequential focal planes that included the cell body and all visible dendrites (Figure 3). The features of the soma and the primary dendritic shaft thickness as well as the branching pattern and spatial orientation of main dendritic shafts in the neuropil supported the classification of CC layer V neurons as VENs. Small adjustments of brightness and background contrast were done in final reconstructed images using Adobe Photoshop CS3 software (Adobe Systems, Inc., United States) without altering the original neuronal features.

Based on the 2D general morphology, we performed the 3D reconstruction of VENs using the Neuromantic free software (v1.6.3 programmed in Borland C++ Builder, University of Reading, United Kingdom). Semiautomatic tracing of the cell body and dendrites was done for the original stack of microscopic images acquired along with the three spatial coordinates. Reconstructions were achieved as a sequence of 3D points with an ASCII-based format representing dendritic trees as a series of connected cylinders of varying radii identified by orthogonal lines from edge to edge (Myatt et al., 2012). The luminosity was inverted to allow more details to be observed in the dendritic shafts contrasting with the background. The contrast was adjusted for the visualization of thin branches. Algorithm and image processing are depicted in Myatt et al. (2012). Final images were saved as SWC + format for storing neuron morphologies (Parekh and Ascoli, 2013). Morphometric data were obtained from the L-Measure free software (Scorcioni et al., 2008) using the 3D reconstructed images. Representative examples of VENs in the CC were studied. Values were calculated for the cell body length, main diameter and volume, dendritic diameter of the primary shafts, total number of branches (i.e., the sum obtained starting from primary dendrites, including segments between branching points, and toward the end of tapered main or collateral branches), and total length and total volume of the dendritic tree.

We obtained 33 VENs that randomly fulfilled the including criteria for study. From our sample, 15 neurons were labeled as VEN 1, 10 were VEN 2, and 5 were VEN 3 (see Results). The number of these Golgi-impregnated VENs per studied case and their location in the CC is shown in Table 2.

**TABLE 2:** Number of Golgi-impregnated VENs in the human cingulate cortex (CC) per studied case.

Case	VEN type 1	VEN type 2	VEN type 3
1	3 [−5.8 mm]		1 [−5.8 mm]
2	4 [−36 mm]		
3	4 [2.7 mm]	3 [2.7 mm]	
4	[−1.3 mm]	7 [−8.3 mm]	4 [4.0 mm]
Total	18	10	5

*Approximate location in the CC for the three types of neurons are indicated in the brackets.*

Afterward, for the 3D reconstruction of dendritic spines, bright-field images were acquired at a final magnification of ×1,300 using an ×100 oil immersion objective lens (plan apochromatic UPlanSApo 1.4 NA, Olympus, Japan). Each image was acquired with high resolution (2070 × 1548 pixels) and submitted to dynamic deconvolution using the Image-Pro Plus 7.0 software. Spines were imaged from proximal to distal branches in each neuron studied. Data were obtained by controlling the focus in the “z” axis and acquiring z-stacks at sequential 0.1 μm steps. Corresponding images were stored as.TIFF files and converted to 8-bit monochromatic pictures. Each spiny dendritic segment imaged consisted of approximately 100–200 sequential frames saved as.TIFF files.

Following Reberger et al. (2018), spines were 3D reconstructed using an algorithm performed in the MATLAB software (R2105b, The MathWorks, United States). That is, after processing the gray-scale slices independently or using median 3D filters in smaller sub-volumes, images were processed using the following steps: (a) outlier removal; (b) edge enhancement using a variant of the “unsharp masking method” and image-filtering approach based on domain transforms (“edge-aware”); (c) binarization using an adaptive thresholding approach; (d) false-positive pruning; (e) 2D flood-fill operation with each slice of the binary volume; (f) tricubic interpolation to smooth transitions between adjacent slices; and (g) visualization of the final volume of the sampled images containing the selected dendritic shafts and their spines (Vásquez et al., 2018) using the “Fiji” ImageJ software (Schindelin et al., 2012) with the “Volume Viewer” plug-in^1^. Images had final adjustments of brightness and contrast made in Photoshop CS3 without altering spine counting or classification.

The identification and classification of each type of 3D-reconstructed dendritic spine was based on previous descriptions (Fiala and Harris, 1999; Arellano et al., 2007a, b; Brusco et al., 2010, 2014; González-Ramírez et al., 2014; Dall’Oglio et al., 2015). By rotating the reconstructed images, spines were inspected at different angles to determine their presence and distribution from proximal to distal dendrites and their number, shape, and size (Reberger et al., 2018). For each spine, we observed (1) the presence, length, and diameter of a neck, (2) the number of protrusions from a single stalk, (3) the head diameter, and (4) the head shape. According to these morphological features, spines were classified as (1) thin, (2) stubby, (3) wide, (4) mushroom-like, (5) ramified, (6) having a transitional aspect between these classes, or (7) “atypical” (or “multimorphic”) spines with usually more complex and varied shapes (Dall’Oglio et al., 2015 and references therein). Small protrusions extending from the head of a spine were classified as spinules (Brusco et al., 2014; Petralia et al., 2018). Spine density was calculated as the number of spines per dendritic length in proximal and distal segments. We counted 55, 104, and 493 dendritic spines in these segments of representative VENs 1, 2, and 3, respectively.

Not all cells were impregnated by the Golgi method. Therefore, descriptive data are provided for representative VENs in layer V of the human CC, but the number of completely impregnated VENs in the ACC and MCC precluded additional extensive statistical analysis. Quantitative data are provided to address how local VENs appear in a *continuum* of morphological features. It has to be mentioned that these morphometric values are not actual ones (as might exist *in vivo*) due to changes in the brain tissue following the *postmortem* period and the various steps of the histological processing (Dall’Oglio et al., 2010, 2013, 2015; Reberger et al., 2018; see also Zeba et al., 2008 for additional discussion). These quantitative data have to be considered with caution and are used as indicators of the relative differences between VENs described here.

All computational procedures were run using Windows Microsoft^®^ (version 10), Intel^®^ Core^TM^ i7-8750H CPU @2.20 GHz, 16.0 GB RAM memory, NVIDIA^®^ GeForce GTX 1051 Ti with 4 GB for image processing.

## Results

Thionin-stained sections served to identify the local cortical cytoarchitectonics and, in the CC, the absence of the inner granular layer IV (Figure 1). Morphological criteria were used to identify CC VENs in layer V. VENs have a typically large and elongated spindle-shaped cell body with two symmetric, vertically oriented primary dendritic shafts (Figure 2). The cell body shape of these cells is similar to those reported by other authors (e.g., Nimchinsky et al., 1995; Allman et al., 2010). All identified VENs in layer V presented the neuronal chromatin aspect and an evident nucleolus. VENs were intermingled with pyramidal neurons and adjacent glial cells (Figure 2). The identification of VENs was reinforced by the Golgi results.

Golgi data provided the shape of VEN dendrites and added new information on spines (Figure 3). In this regard, all studied VENs are spiny neurons. Dendritic spines showed a variety of shapes and sizes. Their types ranged from small to large stubby and wide, thin and mushroom-like, ramified, transitional aspects and/or more complex shapes with multiple bulbous structures, including double spines (i.e., a spine with a neck ending in a bulb, from which a second neck protruded that will end in another bulb; González-Ramírez et al., 2014; Dall’Oglio et al., 2015). These pleomorphic spines were found either isolated or forming clusters, and with different densities in main and collateral dendritic branches. Spinules were observed in different spine types.

**FIGURE 4:**
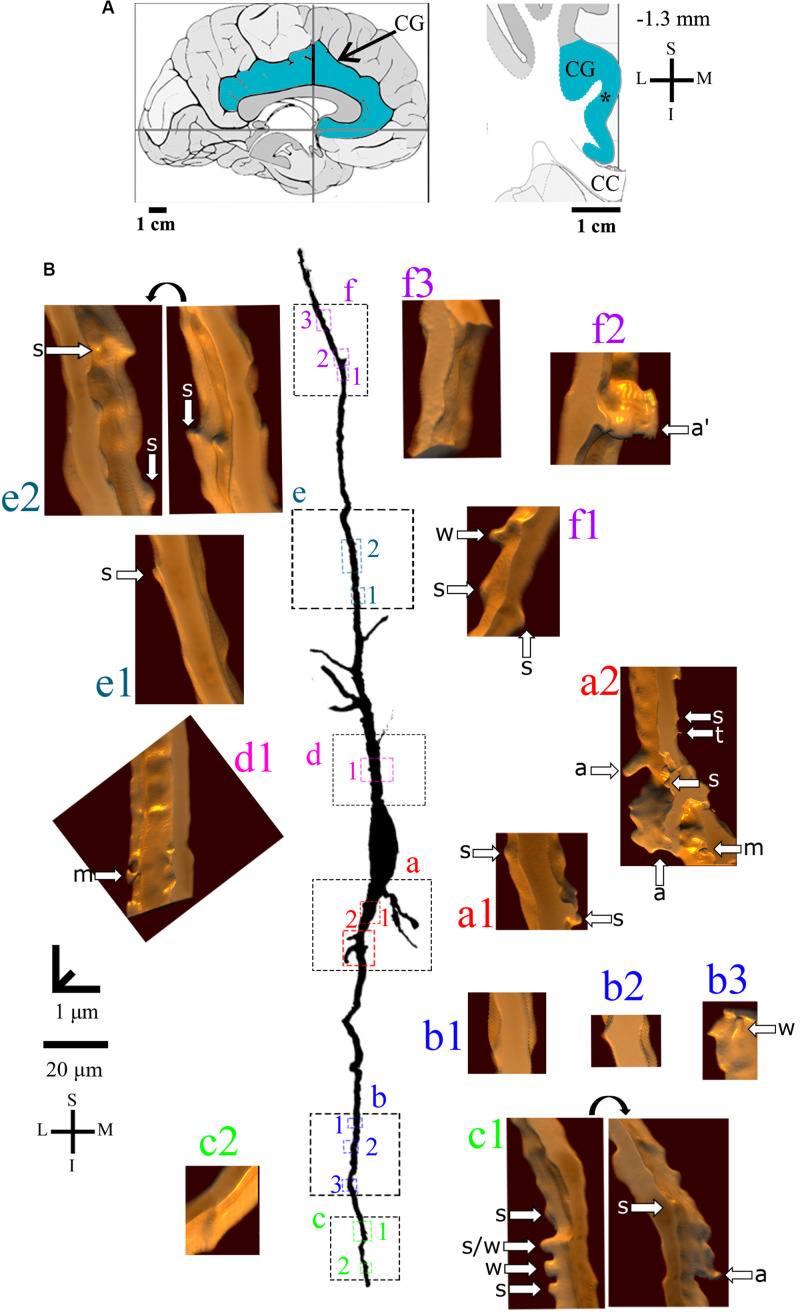
**(A)** Left: Schematic drawing of the medial view of the human brain showing the location of the cingulate gyrus (CG, highlighted in blue), in this case 1.3 mm anterior to the midpoint of the anterior commissure. Right: Higher-magnification drawing of the CG. The asterisk represents the location of the neuron shown in **(B)**. CC, corpus callosum. Adapted from Mai et al. (2008). Coordinates: I, inferior; L, lateral; M, medial; S, superior. **(B)** Two-dimensional (2D, for the general morphology of this neuron) and three-dimensional (3D, for the dendrites and respective spines) reconstructions of serial bright-field photomicrographs of a representative Golgi-impregnated von Economo neuron (VEN 1) in layer V from the human cingulate cortex. The pial surface is at the top. Note the cell body shape and the main ascending and descending dendritic shafts with a straight course and few ramifications. Proximal to distal dendritic segments (identified by colored letters from “a” to “f”) were sampled, and their spines are shown at higher magnification in the adjacent corresponding boxes. Note also the low density of spines and the variety of spine shapes. Spines were classified as stubby (s), wide (w), thin (t), mushroom (m), ramified (r), with a transitional (t/), or atypical aspect (a). Spine types are indicated by arrows after image reconstruction and at different rotating angles. An asterisk with the corresponding spine indicates the presence of a spinule. Image adjustment of contrast made with Photoshop CS3 (Adobe Systems, Inc., United States). Coordinates in **(A,B)**: I, inferior; L, lateral; M, medial; S, superior. Scale = 20 μm for the 2D reconstruction and 1 μm for the 3D reconstructions.

**FIGURE 5:**
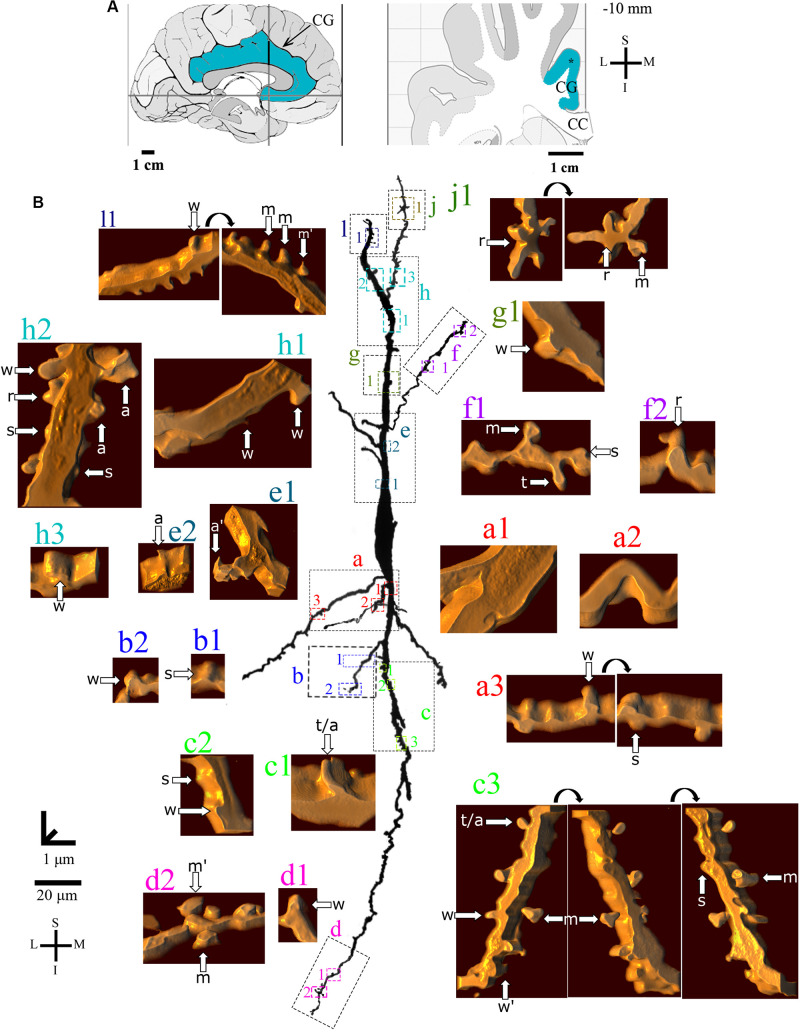
**(A)** Left: Schematic drawing of the medial view of the human brain showing the location of the cingulate gyrus (CG, highlighted in blue), in this case 10 mm anterior to the midpoint of the anterior commissure. Right: Higher-magnification drawing of the CG. The asterisk represents the location of the neuron shown in **(B)**. CC, corpus callosum. Adapted from Mai et al. (2008). Coordinates: I, inferior; L, lateral; M, medial; S, superior. **(B)** Two-dimensional (2D, for the general morphology of this neuron) and three-dimensional (3D, for the dendrites and respective spines) reconstructions of serial bright-field photomicrographs of a representative Golgi-impregnated von Economo neuron (VEN 2) in layer V from the human cingulate cortex. The pial surface is at the top. Note the cell body shape and the main ascending and descending dendritic shafts, but the presence of more collateral ramifications than VEN 1 shown in Figure 4. Proximal to distal dendritic segments (identified by colored letters from “a” to “j”) were sampled, and their spines are shown at higher magnification in the adjacent corresponding boxes. Note also the intermediate density of spines and the variety of spine shapes. Spines were classified as stubby (s), wide (w), thin (t), mushroom (m), ramified (r), with a transitional (t/), or atypical aspect (a). Spine types are indicated by arrows after image reconstruction and at different rotating angles. An asterisk with the corresponding spine indicates the presence of a spinule. Image adjustment of contrast made with Photoshop CS3 (Adobe Systems, Inc., United States). Coordinates in **(A,B)**: I, inferior; L, lateral; M, medial; S, superior. Scale = 20 μm for the 2D reconstruction and 1 μm for the 3D reconstructions.

**FIGURE 6:**
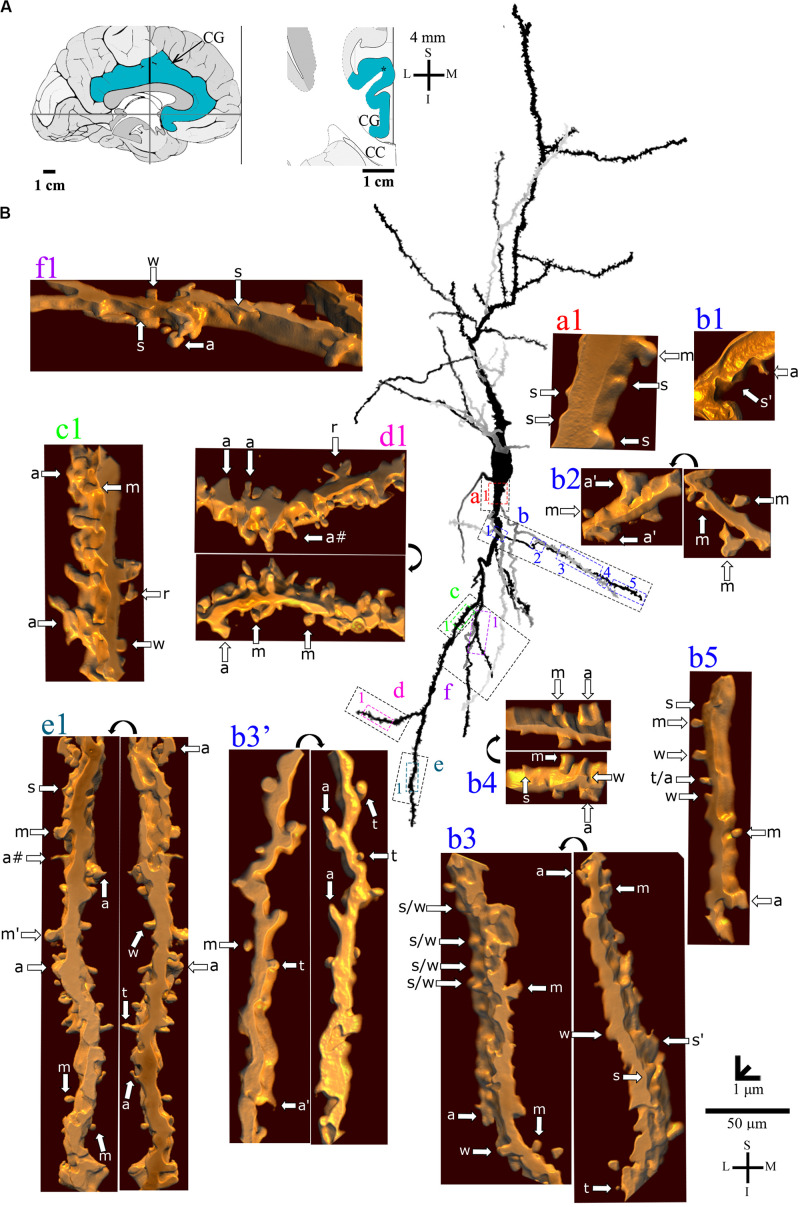
**(A)** Left: Schematic drawing of the medial view of the human brain showing the location of the cingulate gyrus (CG, highlighted in blue), in this case 4 mm posterior to the midpoint of the anterior commissure. Right: Higher-magnification drawing of the CG. The asterisk represents the location of the neuron shown in **(B)**. CC, corpus callosum. Adapted from Mai et al. (2008). **(B)** Two-dimensional (2D, for the general morphology of this neuron) and three-dimensional (3D, for the dendrites and respective spines) reconstructions of serial bright-field photomicrographs of a representative Golgi-impregnated von Economo neuron (VEN 3) in layer V from the human cingulate cortex. The pial surface is at the top. The descending dendritic branch is shown in more detail for the presence, number, distribution, and shape of pleomorphic dendritic spines. Dendrites in gray contrast with dark ones when they are close branches at different focal planes (e.g., b3’ is an upper branch than b3). Proximal to distal dendritic segments (identified by colored letters from “a” to “f”) were sampled, and their spines are shown at higher magnification in the adjacent corresponding boxes. Note the high density of spines with different shapes. Spines were classified as stubby (s), wide (w), thin (t), mushroom (m), ramified (r), with a transitional (t/), or atypical aspect (a). Spine types are indicated by arrows after image reconstruction and at different rotating angles. An asterisk with the corresponding spine indicates the presence of a spinule. a^∗^ represents a double spine. a^#^ represents an atypical spine with a protrusion resembling a filopodium. Image adjustment of contrast made with Photoshop CS3 (Adobe Systems, Inc., United States). Coordinates in **(A,B)**: I, inferior; L, lateral; M, medial; S, superior. Scale = 50 μm for the 2D reconstruction (compare to Figures 4, 5) and 1 μm for the 3D reconstructions.

**FIGURE 7:**
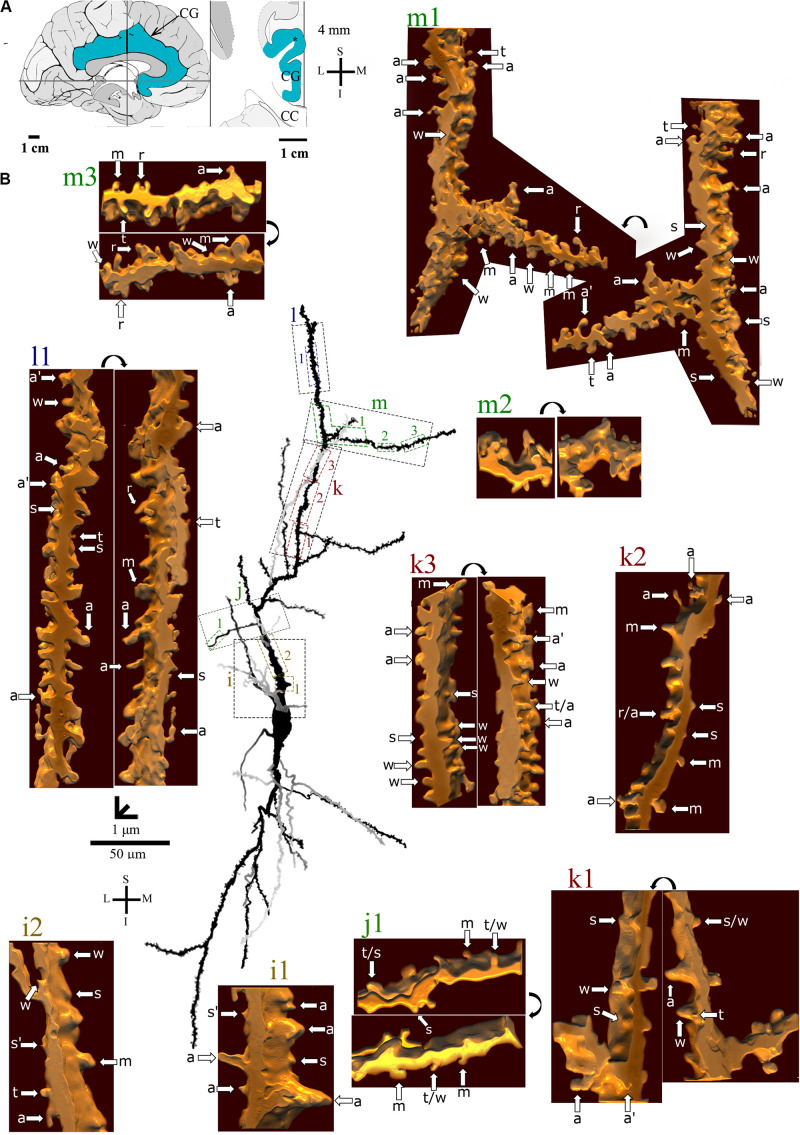
**(A)** Left: Schematic drawing of the medial view of the human brain showing the location of the cingulate gyrus (CG, highlighted in blue), in this case 4 mm posterior to the midpoint of the anterior commissure. Right: Higher-magnification drawing of the CG. The asterisk represents the location of the neuron shown in **(B)**. CC, corpus callosum. Adapted from Mai et al. (2008). **(B)** Two-dimensional (2D, for the general morphology of this neuron) and three-dimensional (3D, for the dendrites and respective spines) reconstructions of serial bright-field photomicrographs of a representative Golgi-impregnated von Economo neuron (VEN 3) in layer V from the human cingulate cortex. The pial surface is at the top. The ascending dendritic branch is shown in more detail for the presence, number, distribution, and shape of pleomorphic dendritic spines. Dendrites in gray contrast with dark ones when they are close branches at different focal planes. Proximal to distal dendritic segments (identified by colored letters from “i” to “m”) were sampled, and their spines are shown at higher magnification in the adjacent corresponding boxes. Note the high density of spines with different shapes. Spines were classified as stubby (s), wide (w), thin (t), mushroom (m), ramified (r), with a transitional (t/), or atypical aspect (a). Spine types are indicated by arrows after image reconstruction and at different rotating angles. An asterisk with the corresponding spine indicates the presence of a spinule. a^∗^ represents a double spine. Image adjustment of contrast made with Photoshop CS3 (Adobe Systems, Inc., United States). Coordinates in **(A,B)**: I, inferior; L, lateral; M, medial; S, superior. Scale = 50 μm for the 2D reconstruction (compare to Figures 4, 5) and 1 μm for the 3D reconstructions.

**FIGURE 8:**
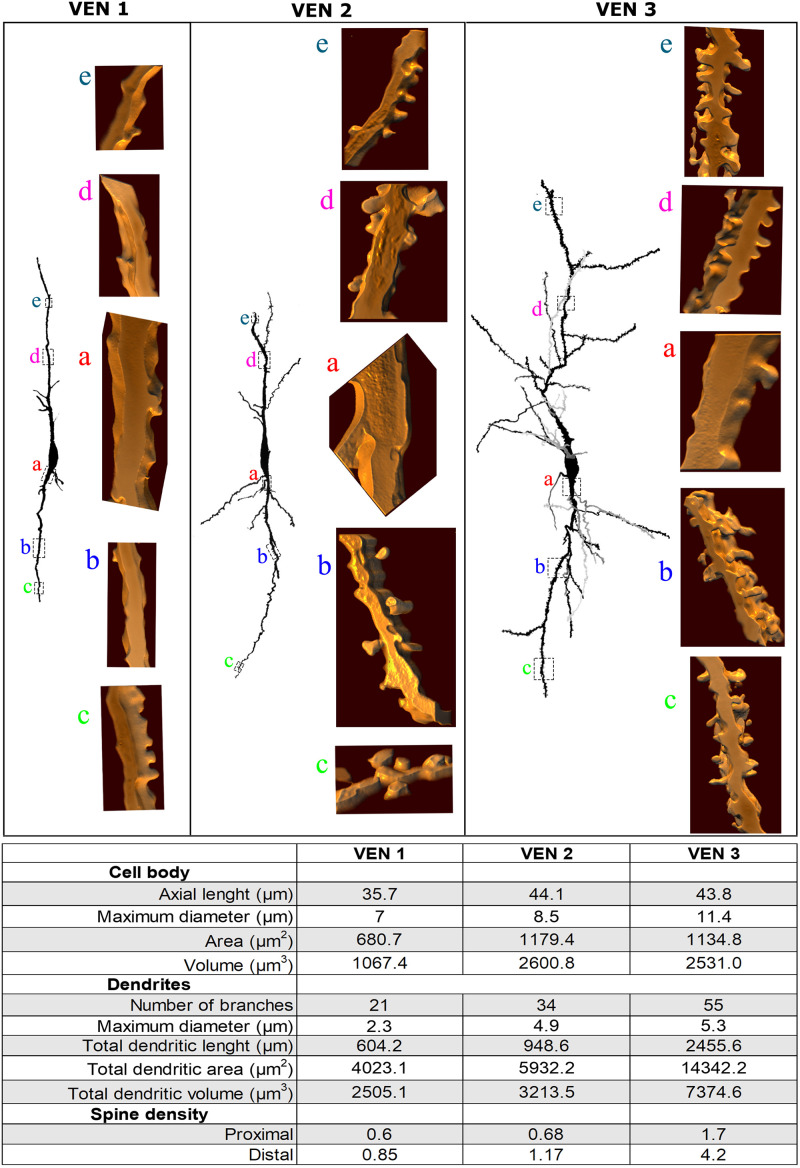
**(Top)** Comparison of the morphological features of spindle-shaped von Economo neurons (VENs) from layer V in the human cingulate cortex. There are heterogeneous dendritic features for the morphological *continuum* of VEN 1 (with few dendritic branches and sparse spines), VEN 2 (with intermediate pattern of dendritic ramification and number of dendritic spines), and VEN 3 (with the highest number of dendritic branching points, collateral branches, and pleomorphic spines). Dendritic spines are shown at corresponding segments to illustrate the differences between these cells. Image adjustment of contrast made with Photoshop CS3 (Adobe Systems, Inc., United States). **(Bottom)** Quantitative data were obtained for the representative VENs 1, 2, and 3 shown above. Morphometrical data were obtained for the cell body and in both ascending and descending dendrites (ranging from lower values in VEN 1 and higher in VEN 3). The density of dendritic spines (number of spines per μm) was calculated along proximal to distal branches (segments indicated in section “Results”). Note fewer dendritic values in VEN 1 and 2 and higher ones in VEN 3.

Our results allowed the identification of a *continuum* of dendritic shapes for VENs in the human CC. We selected representative Golgi-impregnated neurons to exemplify the heterogeneity of their dendritic branching pattern (Figures 4–8 and Supplementary Videos 1–3). There is not a strict separation of VENs into different subtypes at this moment. Rather, the general aspect of these neurons was labeled as VENs 1, 2, and 3 only for easy reference when describing the present data.

The *continuum* of VEN shapes ranged from cells with few dendritic branches and sparse simple spines (VEN 1, Figure 4 and Supplementary Video 1), an intermediate aspect regarding the dendritic branching pattern and the slight increase in the number and types of spines (VEN 2, Figure 5 and Supplementary Video 2), and a more profuse dendritic ramification and the highest density of pleomorphic spines beginning close to the soma and extending through distal dendritic segments (VEN 3, Figures 6, 7 and Supplementary Video 3).

Furthermore, VEN 1 shows both ascending and descending dendrites with a straight course and few ramifications (Figure 4), absence or sparse spines in proximal segments (Figures 4a,d), and a small increase in spine density toward distal segments (Figures 4b,c,e,f). These dendritic spines usually have a simple shape, and most were classified as stubby or wide ones (Figures 4a–f), few as atypical ones with spinule (Figures 4c1,f2). An example of VEN 1 after 3D reconstruction is shown in Supplementary Video 1.

VEN 2 has proximal branching points at both the main ascending and descending primary dendrites (Figure 5). The number of collateral branches is more numerous than VEN 1 but is still limited. The proximal dendritic shafts characteristically show an absence or few spines (Figures 5a1–3,b1,b2,c1,c2) usually with stubby and wide (Figures 5a3,b1,b2) or atypical shapes (Figures 5e1,e2). The presence of intermingled mushroom spines increases in main shaft dendrites from proximal to distal segments (Figures 5c3,d2,h1,j1). This also occurs for transitional/atypical spines (Figures 5c1,c3,e1,h2). Various large stubby or wide spines were found (Figures 5g1,h1–3). Some ramified spines were observed (Figures 5f2,h2,j1). Spinules were present in different spine types (Figures 5c3,d2,e1,l1). An example of VEN 2 after 3D reconstruction is shown in Supplementary Video 2.

VEN 3 displays both high arborization and density of intermingled pleomorphic dendritic spines in main shafts and collateral branches (Figures 6, 7 for the descending and ascending dendrites, respectively). The branching pattern is more profuse and begins close to the cell body in proximal thick branches. Main dendritic shafts maintain the vertical orientation for the ascending and descending branches, whereas the collateral dendrites ramify at different radial angles, including some in a horizontal position (Figures 6, 7). Primary dendrites have various spines, including stubby and wide ones, many atypical aspects, or mushroom shape (Figures 6a1,b1, 7i1,i2), a pattern that also occurs in collateral branches (Figures 6b2–b5, 7j1). The number of pleomorphic dendritic spines increases in intermediate (Figures 6c1, 7k2,k3) and distal segments (Figures 6d1,e1, 7m1–3,l1). There is a myriad of intermingled spines of all sizes and types, i.e., stubby, wide, thin, mushroom, ramified, with transitional shapes, many atypical and complex multimorphic aspects (Figures 6a–h, 7i–n), such as double spines (Figures 6b3,d1, 7m1), and a protrusion resembling a filopodium (Figure 6e1). Pleomorphic spines are found spaced from each other (Figures 6a1,b1,b3’,f1, 7i2,j1,k1) or, more usually, in clusters along different parts of the same dendritic branch (Figures 6c1,d1, 7k3,m1,l1). Spinules were found in different spine types, such as in stubby (Figures 6b1,b3, 7i2), mushroom (Figure 6e1), and atypical spines (Figures 6b3’, 7k1,k3,l1). An example of VEN 3 after 3D reconstruction is shown in Supplementary Video 3.

The heterogeneity for layer V VENs in the human CC is also exemplified by morphometric data of the cell body and spiny dendrites. All VENs have a spindle-like cell body and close values of somatic longitudinal axis length. The number of branches and total dendritic length as well as the density of proximal and distal spines are more abundant in VEN 3 (Figure 8).

## Discussion

Golgi-impregnated VENs show a morphological *continuum* of dendritic and spine features in the human CC. The heterogeneity ranges from few dendritic branches in the main ascending and descending dendritic shafts to a higher arborization with many collateral branches. Differences also occurs for the number, shape, and clustering of dendritic spines from proximal to distal segments. Our results add to the characterization of human CC VENs and have likely implications for the synaptic processing in these cells.

### Morphological Implications

First, there are some inherent difficulties for the study of the human *postmortem* brain tissue that contrast with quickly fixed tissue samples from other species, including anoxic and autolytic disturbances beyond control. However, we do not have evidence to support that VENs 1 and 2 are artifacts of a worst Golgi impregnation of the more complex VENs 3. Nor was the case for the previously published data showing similar VENs 1 in the human ACC (Watson et al., 2006). Axons are not usually visible using the present Golgi technique. This might be due to an insulating myelin sheath that precludes their silver impregnation. Alternatively, axons might not be arising exclusively from the cell body and it might not be easy to identify their emergence in dendrites. It is also worth mentioning that the Golgi method randomly impregnates a few cells at each time per studied sample. This precluded further comparisons of the number of VENs and the dendritic spine density in each one of these VENs along with the human lifespan, the likely interindividual variability, or the existence of sex differences in our available samples. These are possibilities open to being investigated in future research (see also Mann et al., 2011).

The present Golgi data expanded the Nissl staining characterization of VENs by providing additional features and the heterogeneity of spiny dendrites in this particular population of neuron. Our data are discussed in the context of the current morphological description of the human VENs. The referential work of Nimchinsky et al. (1995) depicted VENs with closely related cell body shapes but with a certain degree of variation in the CC. Accordingly, a neuron “was considered a spindle cell on Nissl-stained material if it had an ovoid nucleus… and if it had a basal dendrite that was at least as thick as its apical dendrite… spindle neurons were readily distinguishable from pyramidal neurons and exhibited a variety of morphologies. Some were very slender and elongate, with apical and basal dendrites nearly as thick as the soma at its widest point. Others were shorter, more stout, and usually curved. Occasionally, neurons were encountered with a bifid basal dendrite or a third major dendrite emerging from the soma. In addition, lipofuscin deposits were common and were occasionally so large that they distorted the shape of an otherwise very slender neuron… Although only neurons with a truly spindle-like morphology were considered for this study, it is probable that these represent one end of a spectrum ranging in morphology from the classical pyramid to the most narrow spindle cell. The significance of this cellular variability is not clear, but it might be related to the cytoarchitectonic variability in this region…” (Nimchinsky et al., 1995). We agree with this description. The same possibility for the morphological feature variability of the VEN dendrites and spines was tested here.

VENs would be a subpopulation of pyramidal neurons or “modified pyramidal neurons” (MPN) (Nimchinsky et al., 1995; González-Acosta et al., 2018). Braak (1979) considered layer Vb “slender pyramids or spindles” as MPN in the class of primitively organized Betz-cells in the anterogenual region of the human telencephalon. Banovac et al. (2019) defined “VENs on Golgi staining as a neuron with the following morphological features: an elongated, stick-like cell body gradually continuing into thick apical and basal stem, a brush-like basal stem arborization and an axon origin distant from the cell body.” These authors studied cells in the deep part of layer V and, additionally, in the upper part of layer VI in the human left ACC. Accordingly, other local MPN named “bipolar cells” (although with two primary dendrites, i.e., as multipolar neuron) show an oval soma that is “difficult to distinguish from smaller oval VENs based on the cell body and proximal dendritic morphology… However, … on bipolar modified pyramidal neurons, the prominent basal dendrite is longer, and its thickness decreases gradually without terminal brush-like branching” Banovac et al. (2019). It is still debatable how a “bipolar MPN” with a clear fusiform cell body might differ completely from layer V VENs.

It is worth noting that different nomenclatures would lead to discrepancies in the study of the same nerve cell. For example, Ramón y Cajal (1909-1911) described the morphology of “giant fusiform cells” in the human cingulate gyrus inner “large pyramidal and giant fusiform cell layer” close to “a deep medium-sized pyramidal cell layer.” These “fusiform cells have two dendrites, one of which is very long and seems to ascend to layer 1, whereas the other is sometimes rather long and descends before dividing at acute angles into a number of branches.” This description of giant fusiform cells close to large pyramidal neurons resembles that of layer V VENs at the same time that differs from small fusiform neurons in the inner layer VI (Nimchinsky et al., 1999). On the other hand, although showing a heterogeneous dendritic branching pattern, CC VENs are cells whose morphological aspect could be separated from other populations of cortical MPNs. No image for the various cortical MPNs corresponds exactly to the VENs in layer V as presented here (compared to Figure 2 in Braak, 1980; Figure 6 in Braak and Braak, 1985). These MPNs “deviate substantially from stereotypical pyramidal cells” to include cells with variations in their cellular processes, i.e., the apical dendrite is only a short and very thin process, basal dendrites that do not always have the same diameter and length, and, sometimes, one basal dendrite may be particularly thick and extend in various directions, or cells with various dendrites generated from the lateral surfaces of the soma (Braak, 1980; Braak and Braak, 1985). For example, MPNs from the multiform layer VI of the isocortex emit only two stout dendrites, “one of them is oriented perpendicular to the cortical surface, the other runs in various directions. These cells are therefore referred to as “a pair of compass cells”…with two main dendrites disposed at different angles to each other… The formation of only two main dendrites gives the cell body a triangular or rhombic contour…” (Braak, 1980). The morphological descriptions of MPNs contrast with those for VENs in layer V, their size, and aspects of the cell body shape as well as the orientation and length of the two main primary dendrite shafts.

The criteria for classifying Golgi-impregnated neurons specifically as VENs by Watson et al. (2006) were “an elongated, large soma in layer 5 of the FI or ACC, a prominent basal dendrite, and symmetrical morphology along the horizontal and vertical axes of the cell…” In this regard, there are 29 human neurons labeled as VENs available at the open database “NeuroMorpho.Org”^2^ (version 7.8, released 08/19/2019, content: 112244 neurons). Nine of these reconstructed neurons were from the ACC, and 20 others were from the FI. Three of them show a clear “brush-like” basal dendrite, but 26 others do not display that clearly. No VEN specifically in the ACC shows a prominent brush-like basal dendritic branching in this abovementioned sample. We are thus led to consider that “brush-like” basal dendrites of VENs is one of the possible morphological features for VENs, but it might not be the unique morphological feature for the descending branches. Here, although VEN 3 shows the “brush-like basal stem arborization,” the examples of VENs 1 and 2 do not have the same specific “brush-like” basal dendrites (Figure 8). Otherwise, from the *continuum* of heterogenic shapes of VENs available at NeuroMorpho.org, it would be assumed that 23, 4, and 2 neurons would be VENs 1, 2, and 3, respectively, with similar characteristics as reported here.

### Functional Implications

The human CC VENs compose a salience detection/attentional frontoparietal network, which may modulate complex function as self-awareness and social interpersonal relationships (Cauda et al., 2013, 2014 and references therein). These VEN functional properties rely on the features of dendritic branches and spines for proper synaptic integration, strength, and plasticity. Yang et al. (2019) combined laser-capture microdissection with RNA sequencing and described the transcriptomic profile of VENs from the human ACC. These authors used pyramidal neurons as reference cells and found 344 genes with VEN-associated expression differences related to morphogenesis, including dendrite branching and axon myelination, and human social–emotional disorders (Yang et al., 2019). The laminar distribution of synapses in the human CC suggests that VENs may be modulated by different neurotransmitters. For example, the cells in layer Vb show moderate to high expression of glutamate AMPA, NMDA, kainate, and mGluR2/3 excitatory receptors; GABA_A_ and GABA_B_ inhibitory receptors; adenosine A1 inhibitory receptors; acetylcholine M1 and M3 excitatory receptors, and M2 inhibitory ones; dopamine D1 excitatory receptor; serotonin 2 excitatory receptor; and noradrenalin/adrenalin α1 excitatory and α2 inhibitory receptors (Palomero-Gallagher and Zilles, 2017). Specifically, VENs in the ACC have dopamine D3 and D5 receptors, serotonin-1b and -2b receptors (Watson, 2006), GABA receptor subunit θ, and adrenoceptor α-1A (Dijkstra et al., 2018). Human VENs also characteristically express the activating transcription factor 3 of the CREB protein family likely involved in stress responses and pain sensitivity; interleukin-4 receptor alpha chain linked to inflammatory and allergic reactions; and neuromedin B related to the digestive homeostatic integration, appetite control, gut feelings, and the modulation of appetite with a possible connection of interoception/visceral states with social awareness (Allman et al., 2010; Stimpson et al., 2011; Raghanti et al., 2015). Few human VENs express markers associated with callosal or corticothalamic projections; rather, they prominently express transcription factors of subcerebral projection FEZF2 and CTIP2, which may reach parasympathetic/sympathetic control sites (Cobos and Seeley, 2015). Respectively, the left and the right ACC have been involved with parasympathetic- and sympathetic-associated emotions (Craig, 2005; Cauda et al., 2014; Guo et al., 2016). Our presently described CC VENs could be tested for these neurochemical and functional profiles and compared to other previous data on VENs and MPNs (e.g., Watson et al., 2006; Banovac et al., 2019). Indeed, the “single-section” Golgi method used here was originally developed in rats to be combined with a variety of histochemical procedures (Gabbott and Somogyi, 1984; adapted for humans by Dall’Oglio et al., 2010; but see also Saper, 2005; Zeba et al., 2008).

Human VENs might be considered computationally simple compared to cortical pyramidal neurons, receiving few inputs within individual mini-columns, and likely providing a rapid cortical radial signal transmission (Watson et al., 2006). The aspect of VENs 3, with more dendrites and spines than VENs 1, adds a high level of complexity to this field. More dendrites and varied spines can greatly enhance the computational power of neurons (Spruston et al., 2013; Brunel et al., 2014; Rollenhagen and Lübke, 2016). The use of theoretical models to simulate the electrophysiological dynamics of the heterogeneous human neurons is an alternative to presume the functions of VENs 1–3 at this moment (Brunel et al., 2014). In this regard, the geometry and the functional properties of more branched dendrites affect the linear and non-linear neuronal processing of information (Oakley et al., 2001; Spruston et al., 2013; Brunel et al., 2014; Rollenhagen and Lübke, 2016). Neocortical dendrites in humans can also have distinctive biophysical properties for signal processing that can enhance both synaptic charge transfer from dendrites to soma and spike propagation along the axon (Eyal et al., 2016). In addition, the spine activity-driven changes related to synaptic demand, stability, and plasticity can show region-specific and neuron-specific characteristics (Chen et al., 2011; Dall’Oglio et al., 2015; Berry and Nedivi, 2017; Nakahata and Yasuda, 2018). Spines of different shapes and sizes differ in the membrane surface available for different receptors and their trafficking, the local electrical and biochemical compartmentalization, the degree of cooperativity between adjacent spines, and/or the capacity to disperse second messengers into the parent dendrite. That is, spines can control signaling mechanisms at individual synapses (Bourne and Harris, 2008; Chen et al., 2011; Brusco et al., 2014). In prefrontal pyramidal neurons, the density of dendritic spines shows a developmental pruning and dynamic remodeling in each phase of the reorganization of cortical circuitries along the first decades of the human lifespan, becoming more stable afterward (Petanjek et al., 2011). All these mentioned features of dendritic spines are open avenues for further studies on the morphological and functional interplay of human VENs.

The impact of the structure and the functions of the different spine shapes (stubby/wide, thin, mushroom, ramified, or transitional/atypical) for the fine-tuned synaptic processing were depicted elsewhere (Arellano et al., 2007a; Rochefort and Konnerth, 2012; Yuste, 2013; Stewart et al., 2014; Dall’Oglio et al., 2015; Tønnesen and Nägerl, 2016; Lu and Zuo, 2017; Nakahata and Yasuda, 2018). Large dendritic spines usually are more stable, have a large postsynaptic density, and make strong connections, whereas small spines can be rather transient (Woolfrey and Srivastava, 2016) and/or indicative of connections with a lower resistance to reach the parent dendrite (Segal, 2010). Different spine types were observed along the VEN dendrites. Spines ranged from few spines in dendrites of VENs 1 to a high number of clustered spines of varied shapes and sizes along the dendritic branches of VENs 3. Clustered dendritic spines can modulate the cooperative interaction between neighboring synapses (Yadav et al., 2012) and the network function, thus influencing storage capacity, learning, and memory (Frank et al., 2018). Tiny protrusions identified as spinules were also reliably observed in different Golgi-impregnated spine types in the human ACC. Spinules are functional elements that modulate cellular trans-endocytosis (Spacek and Harris, 2004), representing additional possibilities for neuronal plasticity (Tao-Cheng et al., 2009), even as active zone-free invaginating structures (see further data in Petralia et al., 2018). Therefore, the morphological features of VENs 1–3 are suggestive of different properties for spatial and temporal synaptic processing regulated at every spiny dendritic segment. This would provide additional emergent properties for neural circuitries integrated for complex human information processing, as occurs in the human prefrontal cortex (Petanjek et al., 2011) and for the CC roles on attention, emotion, visceral responses, consciousness, social judgments, cognition, and adaptive behaviors (Allman et al., 2001, 2011a; Butti et al., 2013; Cauda et al., 2013, 2014; Raghanti et al., 2015; Vogt, 2015). However, it is also important to consider that the dendritic spine-free zone of cortical pyramidal neurons develops late in phylogenesis and ontogenesis (Braak, 1980), which suggest particularities and specializations for the excitatory and inhibitory synaptic processing along the dendritic segments of neurons with more or less spines (Peters et al., 1991; Spruston, 2008; Chen et al., 2011; Spruston et al., 2013; Dall’Oglio et al., 2015).

The development of specialized VENs can also bring about intrinsic vulnerabilities (Allman et al., 2001; Butti et al., 2013; Cauda et al., 2014). Proteomic analysis indicated that cytoskeletal dysfunction can be considered an important component of the neuropathology of the major psychiatric disorders involving the human ACC (Beasley et al., 2006). The ACC neurons – specifically the VENs in some cases – are more vulnerable and damaged in cases of the behavioral variant of the frontotemporal dementia and hindered social–emotional functions (Kim et al., 2012; Gami-Patel et al., 2019; Lin et al., 2019); schizophrenia (Krause et al., 2017); suicide in victims with psychotic disorders (Brüne et al., 2011); deficits in understanding non-literal language, humor, and scenes of social interactions related to partial or complete agenesis of the corpus callosum; the autism spectrum and bipolar disorders (Raghanti et al., 2015 and references therein); Alzheimer’s disease (Gefen et al., 2018); altered cardiac vagal tone (Guo et al., 2016); and self-conscious emotional reactivity (Sturm et al., 2013). When further studying VEN shapes and their normal or altered functioning in brain circuitries, it is also important to consider the “…great possibility that interaction between psycho-social environments during brain development results in interindividual differences in brain structure observed later in adult human” (Zeba et al., 2008).

## Conclusion

The human CC shows a *continuum* of morphological features involving the architecture of dendrites and spines of layer V VENs. Our data add to previous morphological studies on the local cytoarchitectonic organization and propose additional functional possibilities for these neurons. The heterogeneity of VENs in the human CC encourages further studies on how these specialized neurons evolved phylogenetically and develop ontogenetically to provide neural computations that, within various neural networks, enhance the complexity and integrate the information processing in the human brain.

## Data Availability Statement

All datasets generated for this study are included in the article/Supplementary Material. Data are public at figshare.com using 10.6084/m9.figshare.11368076.

## Ethics Statement

The studies involving human participants were reviewed and approved by The Brazilian Ethics Committee from the Federal University of Health Sciences of Porto Alegre (UFCSPA; #62336116.6.0000.5345 and 18718719.7.0000.5345). The patients’ next of kin provided written informed consent for brain donation for use in this study. Written, informed consent was obtained from the individuals’ next of kin for the publication of any potentially identifiable data included in this article.

## Author Contributions

NC-J, JR, FF-V, AH, and AR-F: study concept and design, acquisition of data, and elaboration of the manuscript. NC-J and AR-F: two-dimensional reconstructions. JR and AR-F: three-dimensional reconstructions. NC-J, JR, FF-V, and AR-F: interpretation of data. All authors contributed to the article and approved the submitted version.

## Conflict of Interest

The authors declare that the research was conducted in the absence of any commercial or financial relationships that could be construed as a potential conflict of interest.

## Footnotes

1 https://imagej.nih.gov/ij/plugins/volumeviewer.html

2 http://neuromorpho.org

## Supplementary Material

VIDEO S1 Three-dimensional reconstruction of layer V Golgi-impregnated VEN 1 from the human cingulate cortex.

VIDEO S2 Three-dimensional reconstruction of layer V Golgi-impregnated VEN 2 from the human cingulate cortex.

VIDEO S3 Three-dimensional reconstruction of layer V Golgi-impregnated VEN 3 from the human cingulate cortex.
